# Chinese Additive Anti-inflammatory Action for Aortopathy & Arteriopathy (5A) Registry protocol: rationale, design and methodology

**DOI:** 10.1186/s12872-024-03760-y

**Published:** 2024-02-21

**Authors:** Hong Liu, Si-chong Qian, Hai-yang Li, Yong-feng Shao, Hong-jia Zhang, the China Additive Anti-inflammatory Action for Aortopathy, Arteriopathy (5A) Investigators, Hong Liu, Si-chong Qian, Hai-yang Li, Lu Han, Ying-yuan Zhang, Kai Wang, Ying Wu, Liang Hong, Ji-nong Yang, Ji-sheng Zhong, Bing-qi Sun, Xiao-cheng Liu, Dong-kai Wu, Guo-liang Fan, Jun-quan Chen, Sheng-qiang Zhang, Yi-yao Jiang, Xing-xing Peng, Zhi-hua Zeng, Xin Zhao, Peng-cheng Tang, Xiao-yan Feng, Cheng-bin Tang, Hui-jun Zhang, Zhan-jie Lu, Si-qiang Zheng, Chen Zhang, Yong-feng Shao, Hong-jia Zhang, Peng-cheng Zhu, Hong-hua Yue, Ling-chen Huang, Feng Wu, Xiao-han Xu, Xiao-hu Lu, Wei-dong Gu

**Affiliations:** 1 Department of Cardiovascular Surgery, the First Affiliated Hospital of Nanjing Medical University, No. 300 Guangzhou Road, Nanjing, 210029 China; 2 Department of Cardiovascular Surgery, Beijing Anzhen Hospital, Capital Medical University, No. 2 Anzhen Road, Chaoyang District, Beijing, 100029 China

**Keywords:** Acute aortic syndrome, Inflammation, Systemic inflammatory response syndrome

## Abstract

**Background:**

Acute aortic syndrome (AAS) is a life-threatening condition. Inflammation plays a key role in the pathogenesis, development and progression of AAS, and is associated with significant mortality and morbidity. Understanding the inflammatory responses and inflammation resolutions is essential for an appropriate management of AAS.

**Method:**

Thirty Chinese cardiovascular centers have collaborated to create a multicenter observational registry (named Chinese Additive Anti-inflammatory Action for Aortopathy & Arteriopathy [5A] registry), with consecutive enrollment of adult patients who underwent surgery for AAS that was started on Jan 1, 2016 and will be ended on December 31, 2040. Specially, the impact of inflammation and anti-inflammatory strategies on the early and late adverse events are investigated. Primary outcomes are severe systemic inflammatory response syndrome (SIRS), multiple organ dysfunction syndrome (MODS), Sequential Organ Failure Assessment (SOFA) scores at 7 days following this current surgery. Secondary outcomes are SISR, 30-day mortality, operative mortality, hospital mortality, new-onset stroke, acute kidney injury, surgical site infection, reoperation for bleeding, blood transfusion and length of stay in the intensive care unit.

**Discussion:**

The analysis of this multicenter registry will allow our better knowledge of the prognostic importance of preoperative inflammation and different anti-inflammatory strategies in adverse events after surgery for AAS. This registry is expected to provide insights into novel different inflammatory resolutions in management of AAS beyond conventional surgical repair.

**Trial registration:**

ClinicalTrials.gov Identifier: NCT04398992 (Initial Release: 05/19/2020).

## Introduction

Aortopathy represent a major clinical challenge and are regarded as one of the leading causes of mortality among cardiovascular disorders [1]. However, the pathological mechanisms underlying aortopathy are still far from being well understood, which makes treating this life-threatening challenging [2]. It is increasingly clear that inflammation plays a key role in the development and progression of acute aortic syndrome (AAS) independent of cholesterol and other traditional risk factors, and characterizes both systemic and local condition [3, 4].

Currently, surgery is considered the best treatment option for patients with AAS. In addition to systemic inflammatory responses triggered by AAS itself [5], however, procedural factors including surgical trauma, anesthesia, cardiopulmonary bypass, hypothermia, circulatory arrest, and blood transfusion as well as mechanical ventilation initiated a cascade of inflammation, which further exacerbates “inflammatory storm”, and is associated with significant postoperative mortality and morbidity [6]. Along with surgical evolutions, scientists have made new discoveries and achievements in the underlying mechanism and understanding of inflammation of AAS, which greatly encourage us to optimize treatment for these patients. Going beyond traditional surgery, anti-inflammatory action is crucially important to target the residual cardiovascular risk by specific anti-inflammatory interventions as a crucially adjunct therapeutic strategy to improve the well-being of patient [7].

A better understanding of the interaction between patient’s inflammatory responses and anti-inflammatory strategies which may limit the residual cardiovascular risk is essential for the development of novel preventive, diagnostic, and therapeutic approaches, providing a critical pathophysiological insight into the role of inflammation in risk assessment and anti-inflammatory targeting [8, 9]. The epidemiological observation that biomarkers of inflammation are associated with clinical cardiovascular risk supports the theory that targeted anti-inflammatory treatment appears to be a promising strategy in reducing residual cardiovascular risk on the background of traditional surgical repair as well as basic therapy [10]. Previous researches have shown that ulinastatin used in cardiac surgery may be effective in prevention of cardiovascular events through an anti-inflammatory effect [11, 12]. This residual inflammatory risk has increasingly become a viable therapeutic targeting on the background of validated surgical repair as well as basic medical therapy for AAS.

Although aortic dissection registries have been established during the last years, such as the International Registry of Acute Aortic Dissection (IRAD) [13], the Nordic Consortium for Acute Type A Aortic Dissection (NORCAAD) Registry [14], German Registry for Acute Aortic Dissection type A (GERAADA) [15], the Society of Thoracic Surgeon (STS) database [16], and European Registry of Type A Aortic Dissection (ERTAAD) [17], there are currently no dedicated registry to prospective collections and characteristics of inflammatory responses, anti-inflammatory strategies, and clinical outcomes especially for AAS patients. We have established a multicenter research collaboration (named “Chinese Registry of Additive Anti-inflammatory Action for Aortopathy & Arteriopathy [5A]”) and planned a prospectively observational study to understand the patient’s inflammatory responses, characterize the potential anti-inflammatory strategies, and evaluate clinical outcome and prognosis of AAS patients at 15 years in a large study of Chinese population.

### Study objective

The objectives of the study are:

- To understand the epidemic trends, contemporary therapeutic strategies, and clinical outcomes in China.
- To identify risk factors associated with poor prognosis and to determine whether there is a relationship between therapeutic strategy and prognosis.
- To explore novel inflammatory markers that provided insight into early detection, early diagnosis, and early warning of disease deterioration.
- To evaluate perioperative treatment responses and the short, mid and long -term outcomes of AAS.
- To develop risk assessment or prediction tools that can be used for early warning and proper intervention selection and to improve prognosis for patients using artificial intelligence methods.
- To facilitate attention and interest on inflammatory responses and anti-inflammatory strategies in AAS.
- To describe the demographic and socioeconomic characteristics, comorbidities, and symptom severity of patients in Chinese hospitals.
- To detail the diagnosis, treatment, and procedures as well as critical care of AAS during hospitalization.
- To investigate the development, the progression, prognosis or outcome of AAS patients.

## Methods and analysis

### Study design

**Fig. 1:**
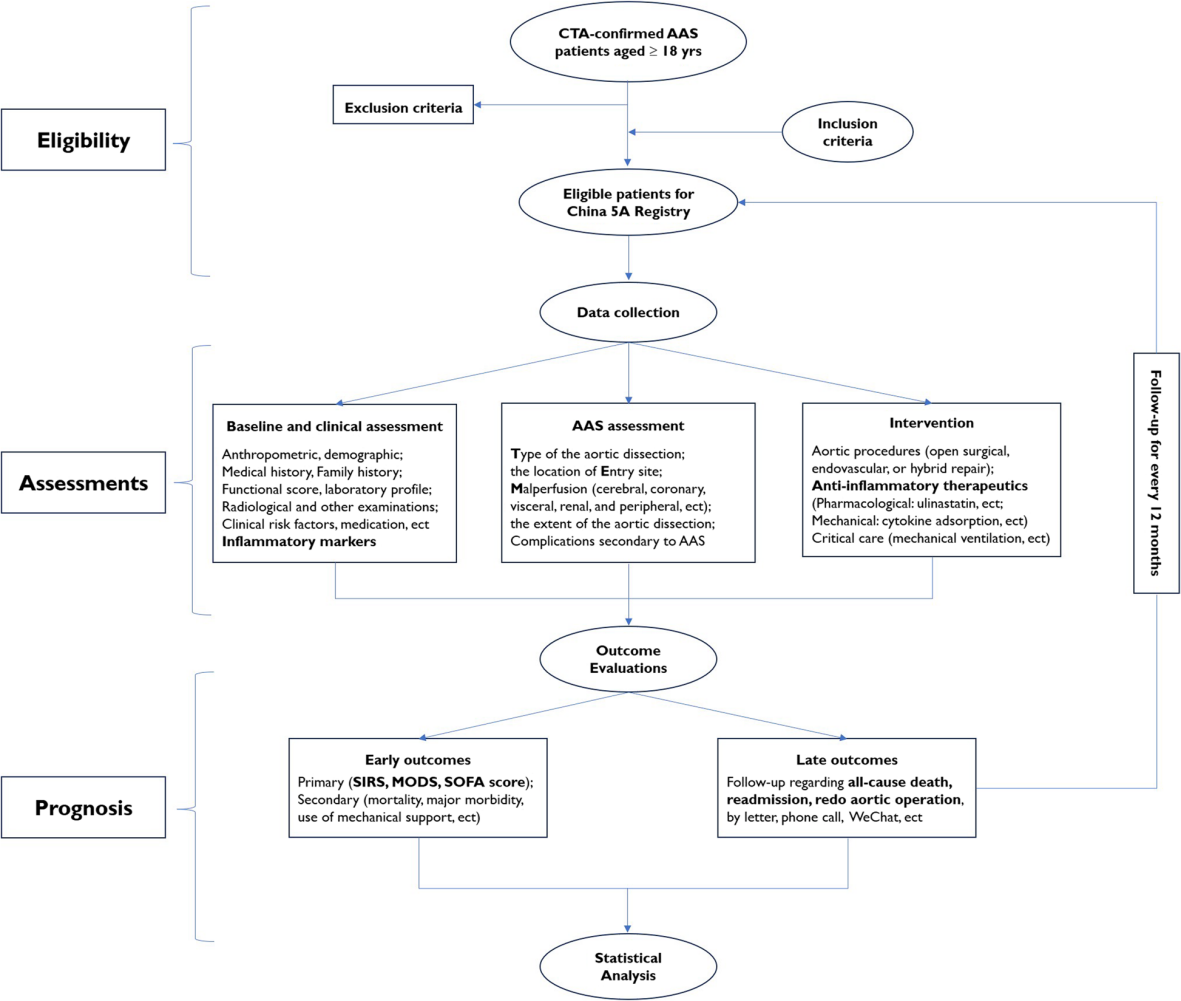
The flow chart of China 5A Registry. *AAS* Acute aortic syndrome, *5A* Additive Anti-inflammatory Action for Aortopathy & Arteriopathy, *SIRS* Systemic inflammatory response syndrome (SIRS), *MODS* multiple organ dysfunction syndrome, *SOFA* Sequential Organ Failure Assessment

This investigator-initiated study is a prospective, ongoing, national, multicenter, registry-based, real-world study with consecutive enrollment of adult patients who underwent surgery for AAS that was started on Jan 1, 2016 and will be ended on December 31, 2040 in more than Chinese 20 hospitals. This registry is the first program specifically designed to investigate the inflammatory responses and anti-inflammatory strategies for AAS. Data management and analysis were performed by the 5A investigators. The study design and flowchart are depicted in Fig. 1.

### Ethics and trial registration

This present study was conducted in accord with the Declaration of Helsinki and registered in Clinical Trials. gov with No. NCT04398992. The Institutional Review Board of Beijing Anzhen Hospital of Capital Medical University and Nanjing Medical University approved this study (first version 1.0, KL20153256, updated version 2.0, No. 2021-SR-281). Patient written consent for the publication of the study data was waived due to this retrospectively observational study. The paper complies with the SPIRIT guidelines for study protocols adapted for designing and reporting registry [18].

### Setting

**Fig. 2:**
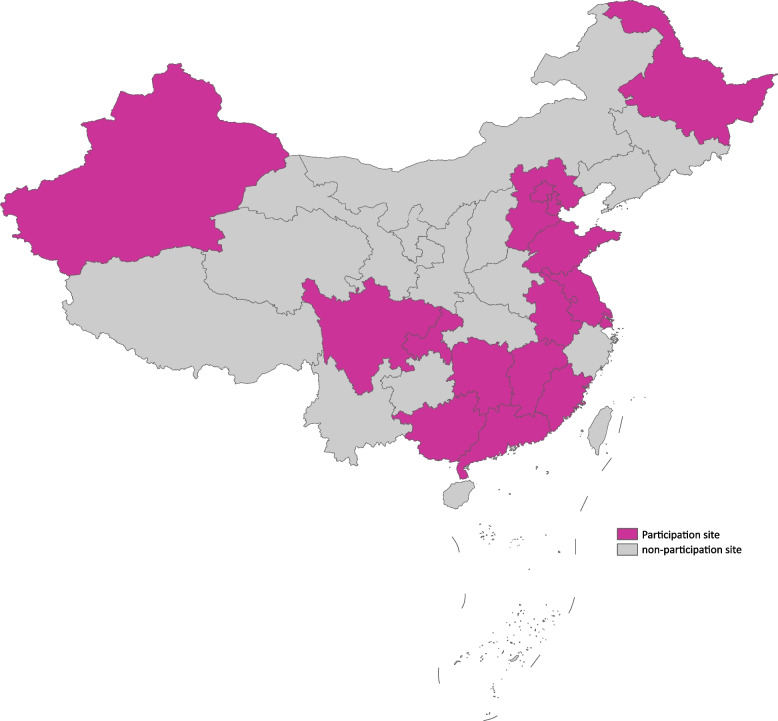
Geographic distribution of the institutions participating China Additive Anti-inflammatory Action for Aortopathy & Arteriopathy (5A) Registry

Participating site inclusion criteria is an acute care hospital providing 24/7 emergency care for AAS. Enrollment of patients was started in January 2016 and will end in December 2040. As 31 December of 2023, this study has been conducted at approximately 30 hospitals across China (Fig. 2), and the number of hospitals will be increased in case more hospitals continue to participate into this Chinese 5A Registry in the future.

### Patient and public involvement

Patients with AAS are recruited from multiple centers across China. In the course of the study, the subjects’ personal privacy and data confidentiality are protected and health managers are specified to maintain further contact with the patient. The feedbacks of patients are also be regularly recorded into the system. Neither participants nor the public has been or will be involved in the process of designing, conducting, or reporting this study.

### Target population and diagnostic criteria

Aortopathy and arteriopathy are diagnosed based on the International Classification of Diseases (ICD)-10th revision. AAS is diagnosed by computed tomography angiography (CTA), and is classified according to the Stanford system: type A involves the ascending aorta, regardless of the site of the primary intimal tear, and type B involves only the descending aorta [19]. Inclusion, exclusion and withdrawn criteria are showed in Table 1.

**Table 1:** Inclusion, exclusion and withdrawn criteria

**Inclusion criteria**
– Aged 18 years or older
– Patients with diagnosis of AAS confirmed by CTA, including aortic dissection, penetrating aortic ulcer or intramural hematoma
– Symptoms started within 14 days from surgery
– Patients received medical therapy, open surgical, endovascular, or hybrid repair
– Any other major cardiac surgical procedure concomitant with surgery for AAS, such as coronary artery bypass grafting or carotid artery replacement;
– The patient or guardian agrees to participate in this study
**Exclusion criteria**
– Patients aged < 18 years
– Onset of symptoms > 14 days from surgery
– AAS secondary to traumatic or iatrogenic injury
– Patients who declined participation in registration and follow-up investigation
**Withdrawn criteria**
-Patients presenting with one or more of the following conditions will be withdrawn from this study:
-The patient dissatisfies the inclusion criteria
-The patient withdraws the informed consent form
-Any situation where the investigators believe that the patient should discontinue from this study for safety reasons or conflict of interest of the patient

*AAS* Acute aortic syndrome, *CTA* Computed tomography angiography

### Data collection and definition criteria

Information including but limited to anthropometrics, demography, medical history, family history, clinical risk factors, medication, treatment strategies, critical care pattern, postoperative outcomes, and prognosis and follow-up information are collected after the patient is admitted to emergency department or department of cardiovascular surgery in each hospital.

- Anthropometric and demographic characteristics: age, sex, race, height, weight, body mass index, and body surface aera, ect.
- Baseline clinical assessments: clinical features, vital signs, physical examination, time from symptom onset to operation, medical history (smoking, drinking, systemic hypertension, pulmonary hypertension, diabetes mellitus, hypercholesterolemia, stroke or transient ischemic attack, chronic pulmonary disease, chronic renal disease [20], etc.), family history, genetic syndrome (Mafan syndrome, Loeys–Dietz syndrome, Ehlers–Danlos syndrome, and Turner syndrome, ect [21]), etc.)
- Critical preoperative state: Preoperative cardiac shock, resuscitation, inotropes, and ventilation before anesthetic room. These conditions will be reported as separate variables.
- Functional scores: Acute Physiology and Chronic Health Evaluation II scores, NYHA class, EuroScore, Glasgow Coma Scale Score, etc.
- Laboratory profiles: complete blood count, biochemistry, blood-gas test, coagulation routine, myocardial markers, and myocardial enzyme profiles, etc.
- Inflammatory biomarkers: C-reactive protein, interleukins, tumor necrosis factor-alpha, ect.
- Instrumental examinations: CTA, electrocardiography, echocardiography, magnetic resonance imaging, X-chest, gastrointestinal endoscope, and bronchoscopy, etc.
- Medication use at last month before surgery: antidiabetic, blood-lowering, or antithrombotic drugs (exposure to any of the following antithrombotic drugs within one week before surgery: aspirin, clopidogrel, ticagrelor, prasugrel, ticlopidine, heparin, fondaparinux, direct oral anticoagulants and/or warfarin). All concomitant medications will be documented with generic drug name (INN) or trade name, with start and stop dates.

In particular, malperfusion refers to acute organ ischemia secondary to aortic branch vessel hypoperfusion. This severe condition is usually classified based on clinical signs and symptoms [22, 23]. Attention is paid to the information of AAS: type of the dissection, extent of the dissection, and malperfusion (cerebral, coronary, visceral, renal, and peripheral), as well as the location of entry site.

#### General

- Malperfusion is defined as clinical or biochemical evidence of end-organ dysfunction and required radiographic evidence of arterial obstruction of the requisite vascular bed [24].

#### Specific

- Cerebral malperfusion is defined as clinical evidence of focal or generalized neurologic deficit involving the brain, presenting with acute stroke.
- Spinal malperfusion is defined as clinical evidence of focal or generalized neurologic deficit involving the spine, presenting with paraparesis/paraplegia.
- Coronary or myocardial malperfusion is defined as evidence of any changes in ST level in electrocardiogram, and or an increase in cardiac enzymes such as troponin or creatine kinase indicative of myocardial ischemia.
- Visceral or mesenteric malperfusion is defined as sudden, mild-to-severe abdominal pain with elevated lactate, signs of peritonitis, elevated liver enzymes, with or without nausea and vomiting, which is accompanied or not by rectal bleeding or bloody diarrhea [25].
- Radiographic renal malperfusion is defined on radiographic grounds involving asymmetric contrast enhancement secondary to static or dynamic obstruction of any renal vessel or any renal vessel coming off the false lumen.
- Clinical renal malperfusion is defined as evidence of biochemical or clinical criteria with elevated creatinine levels or decreased urine output.
- Peripheral malperfusion is defined as loss of pulse with or without sensory or motor deficits of any limb.
- Other definitions and terminologies refer to the Society of Thoracic Surgeons database [26].

## Intervention and procedure

### Surgical repair

- Timing of surgery which is classified in five categories based on increasing severity of hemodynamic instability and the need and timing of cardiopulmonary resuscitation: urgent, emergency grade 1, emergency grade 2, salvage grade 1, and salvage grade 2 [17].
- Aortic surgery: open surgical repair (hemiarch, partial arch, and total arch replacement), endovascular repair, or hybrid repair. The choice of technique is primarily determined by comprehensive consideration of the condition of the individual, characteristics of the dissected aorta, and the surgeon's preferences and experiences [27].
- Procedure-related variables: Main surgical procedure and concomitant procedures, cannulation site, nasopharyngeal and rectal temperature, duration of Unilateral antegrade cerebral perfusion time, Cross clamp time, myocardial ischemia, cardiopulmonary bypass, hypothermic circulatory arrest, Rewarming time, and cerebral perfusion mode will be collected.

Other aspects of clinical care such as medication (antihypertensive, antibiotic, and lipid-decreasing drug, ect.) is at the discretion of the local physicians and are based on the Chinese and international guidelines or expert consensus.

### Intraoperative findings

- Data on the anesthetic profiles, including American Society of Anesthesiologists Physical Status (ASA-PS), anesthetic approach, anesthetic drugs, and duration;
- Data on the intraoperative findings of the pericardium, ascending aorta and aortic arch will be collected. Importantly, the location of entry site, the extent of aortic dissection at the level of the Valsalva sinuses and morphology of the aortic valve will be described.

### Anti-inflammatory therapeutics

#### Pharmacological

The details including dosage, administration approach, frequency of use, maintenance time, and adverse reactions as well as manufacturer are collected (ulinastatin, Xuebijing, Phlegmyheatclear, statin, and colchicine, ect).

#### Mechanical

The details including administration approach, use duration, and adverse reactions as well as manufacturer are collected (cytokine adsorption, ultrafiltration during CPB, and leukocyte depletion, ect).

All concomitant pharmacological medications and mechanical devices will be documented with trade name, with start and stop dates.

## Outcome and definition criteria

### Primary outcome

#### Severe systemic inflammatory response syndrome (SIRS)

Following the International Pediatric Sepsis Consensus: Definitions for Sepsis and Organ Dysfunction in Pediatrics, SIRS was defined as the presence of at least 2 of the 4 age-specific criteria: temperature, heart rate, respiratory rate, and leukocyte count, one of which must be abnormal temperature or leukocyte count. severe SIRS was defined as meeting all 4 aforementioned criteria, measured immediately following surgery through postoperative day 7, hospital discharge, or death, whichever occurred first [28, 29].

#### Multiple organ dysfunction syndrome (MODS)

MODS is defined as dysfunction of two or more organs (involving the respiratory, cardiovascular, renal, hepatic, gastrointestinal, hematological, and central nervous system) following surgical repair, measured immediately following surgery through postoperative day 7, hospital discharge, or death, whichever occurred first [30, 31].

#### Sequential Organ Failure Assessment (SOFA) scores

The mean daily Sequential Organ Failure Assessment (SOFA) score while the patient was in the ICU, as measured since ICU admission immediately after surgery to a maximum of 28 days. The daily SOFA score after surgery was calculated for each patient on the basis of six organ systems: cardiovascular, neurologic, respiratory, renal, hepatic, and coagulation systems. (Scores for each system range from 0 to 4, with higher scores indicating more severe organ-system dysfunction; maximum score, 24) [32, 33]. Daily scores were totaled for each patient’s ICU stay and divided by the number of days that they remained in the ICU in order to calculate the mean SOFA score for that patient.

### Secondary outcome

-Mortality: 30-day mortality, operative mortality, ICU mortality, and in-hospital mortality during the index hospitalization.

Of note, operative mortality, defined as any death, regardless of cause, occurring within 30 days after surgery in or out of the hospital and after 30 days during the same hospitalization subsequent to the operation according to The Society of Thoracic Surgeons criteria [34].

-Major morbidity: neural (stroke, paraplegia, and hemiplegia, ect), renal (major acute kidney event [defined as death, new requirement for renal-replacement therapy, or sustained renal failure [stage 2 or 3 acute kidney injury] at 30 days]) [35], visceral (liver failure, gastrointestinal bleeding, and mesenteric ischemia, ect), cardiovascular (myocardial infarction, ventricular arrhythmia, acute heart failure, refractory hypotension, pericardial tamponade, distal aneurysm rupture, and shock, ect), pulmonary (pulmonary edema, tracheotomy, pulmonary hypertension, severe pulmonary infection, acute respiratory distress syndrome, and re-intubation, ect), infection (sternal infection, mediastinitis, bacteremia, septicemia, and sepsis, etc.), bleeding (fatal bleeding, intracranial bleeding, bleeding with a hemoglobin decrease of > 5 g/dL [3.1 mmol/L], and bleeding requiring reoperation).

#### Mechanical support

Perioperative use of intra-aortic balloon pump (IABP), extracorporeal membrane oxygenation (ECMO), ventricular assist device (VAD), continuous renal replacement therapy (CRRT), and artificial liver support system (ALSS) for various comorbidities as appropriate, and the duration of these treatments will be documented.

-Time endpoint: length of stay of mechanical ventilation, ICU, and hospital duration.

## Late outcomes

Data on patient’s survival status will be collected. Patients lost to follow-up will be reported. Information regarding all-cause death, readmission, and repeat cardiovascular procedures regardless of surgical, endovascular, or hybrid repair will be reported along with its urgency, indications and aortic segment treated. Routine follow-up was carried out routinely every 12 months by letter, phone call, contact with the patients’ primary doctor, or review of medical records.

### Harms

No harmful events will occur during data collection because of the nature of an observational study without interventions.

### Sample size calculation

According to the sample size design scheme of this register study, the sample size has been taken to be about 20 times of the number of independent variables [17]. About 200 baseline, clinical and procedural and postoperative outcome as well as follow-up variables are expected to be included in the analysis. Taking into account a lost-to-follow-up and culling rate of 10%, estimated number of patients to be included in the study is 4500.

### Statistical analysis

Continuous variables will be reported as means ± standard deviation (SD) or as medians (interquartile ranges [IQR]) and compared using Student’s T test and Mann–Whitney test or Kruskall-Wallis test if appropriate. Categorical variables will be stated as absolute and relative frequencies and compared using the χ2 test or Fisher’s exact test if appropriate. Missing data will be handled with multiple imputation as appropriate. Statistical tests are considered significant when the two-sided *P* value is < 0.05.

Differences in primary and secondary outcomes between groups such as the proportion of SIRS and MODS at 7 days, and the occurrence of all-cause mortality at 30 days and ICU discharge and hospital discharge will be compared using binary logistic regression, measured by odd ratios (OR) with their 95% confidence interval (CI). The analysis will be adjusted for confounders, including anthropometric and demographic features, baseline clinical assessments, critical preoperative state, functional scores, laboratory profiles, inflammatory biomarkers, imaging examinations, and medication use. Linear regression will be used to evaluation the relationship between these predictors and continuous outcomes (such as SOFA score), measured by regression coefficient (β) with their 95% CI.

Analysis of time-to-event will be conducted using Kaplan–Meier method and compared with log-rank test. Differences in time-to-event will be compared using Cox proportional hazards method with hazard ratios (HR) with their 95% CI. Competing risk analysis with the Gray’s test will be performed for late non-fatal adverse events because patient’s death might hinder the observation of these events, in which risk estimates will be reported as sub-distribution HR with their 95% CI.

Sensitivity analysis will test the robustness of these outcomes in complete data and imputation data. The outcomes will be further analyzed by the following subgroups: (1) sex (male vs female), (2) age (< 60 vs > 60 yrs), (3) BMI (< 25 VS > 25 kg/m2), (4) timing of surgery (urgent, emergency, or selective), (5) aortic surgery (proximal vs extensive repair), and (6) inflammatory responses (hyperinflammatory vs hypoinflammatory), and so on.

## Discussion

So far, there has been a few researches on inflammation of aortopathy and arteriopathy in national and international level and reports on the anti-inflammatory therapy are limited. In China, there is a lack of multicenter research on aortopathy and arteriopathy population and most studies are retrospective type. So, a relatively perfect national population registration research cohort database may be beneficial. This study, in collaboration with the Chinese AAS collaboration Database, aims to optimize anti-inflammatory strategies and develop new anti-inflammatory approaches for patients with AAS. This will help provide evidence-based medical evidence for the inflammation resolutions of patients and the selection of related techniques.

Major innovations of our study are: First, this is the first large-scale prospective multi-center registry study to focus on inflammation and anti-inflammatory strategies in the cardiovascular field, especially for aortopathy and arteriopathy. Second, we apply artificial intelligence combination with multi-omics analysis and bioinformatics to make new scientific discoveries such as risk prediction constructions, to improve the accuracy of evaluation of morbidity and mortality for aortopathy and arteriopathy. Furthermore, we investigate the inflammation-based risk factors influencing the prognosis and investigate the interplay between inflammation and anti-inflammatory strategies. individualized treatment effect prediction will advance our better knowledge of treating the right patients, which help guide shared decision-making by discriminating patients who benefit most from anti-inflammatory treatment, to improve clinical outcome.

However, this study has several limitations. In terms of sample selection, many patients with AAS who admitted to department of vascular surgery will not be included in the study. Another limitation could be selection bias due to involvement of researchers from various centers and broad selection criteria. In terms of inclusion and exclusion criteria, the conditions are relatively broad, and more interference factors are not considered. In terms of prognostic evaluation, there are few indicators to be examined, and detailed cognitive evaluation will not be done in the follow-up.

## Conclusion

This Chinese 5A registry will advance our better knowledge of prognostic importance of perioperative inflammation and different anti-inflammatory strategies in morbidity and mortality fowling AAS surgery, which is expected to provide insights into novel inflammatory resolutions to improve the operative survival and prognosis of patients with AAS beyond conventional surgical repair as well as basic medical treatment. Nevertheless, future research detailing the inflammatory pathways and their link with AAS as well as chronic inflammatory disorders are also wanted to explore etiological and pharmacological therapeutic strategies.

## Data Availability

The datasets used and analyzed during the current study are available from the corresponding author upon reasonable request. This project is an observational cohort study with no expected adverse events. Standard follow-up training is required, and clinicians and health managers are required to network with patients.
